# *TreeSimGM*: Simulating phylogenetic trees under general Bellman–Harris models with lineage‐specific shifts of speciation and extinction in R

**DOI:** 10.1111/2041-210X.12917

**Published:** 2017-11-13

**Authors:** Oskar Hagen, Tanja Stadler

**Affiliations:** ^1^ Swiss Federal Research Institute WSL Birmensdorf Switzerland; ^2^ Landscape Ecology Institute of Terrestrial Ecosystems ETH Zurich Zurich Switzerland; ^3^ Department of Biosystems Science and Engineering ETH Zurich Basel Switzerland; ^4^ Swiss Institute of Bioinformatics (SIB) Lausanne Switzerland

**Keywords:** birth–death model, macroevolution, phylogeny, simulation

## Abstract

1. Understanding macroevolutionary processes using phylogenetic trees is a challenging and complex process that draws on mathematics, computer science and biology. Given the development of complex mathematical models and the growing computational processing power, simulation tools are becoming increasingly popular.
2. In order to simulate phylogenetic trees, most evolutionary biologists are forced to build their own algorithms or use existing tools built on different platforms and/or as standalone programmes. The absence of a simulation tool accommodating for user‐chosen model specifications limits, amongst others, model testing and pipelining with approximate Bayesian computation methods or other subsequent statistical analysis.
3. We introduce *“TreeSimGM*,” an r‐package simulation tool for phylogenetic trees under a general Bellman and Harris model. This package allows the user to specify any desired probability distribution for the waiting times until speciation and extinction (e.g. age‐dependent speciation/extinction). Upon speciation, the user can specify whether one descendant species corresponds to the ancestor species inheriting its age or whether both descendant species are new species of age 0. Moreover, it is possible to scale the waiting time to speciation/extinction for newly formed species. Thus, *“TreeSimGM”* not only allows the user to simulate stochastic phylogenetic trees assuming several popular existing models, such as the Yule model, the constant‐rate birth–death model, and proportional to distinguishable arrangement models, but it also allows the user to formulate new models for exploration. A short explanation of the supported models and a few examples of how to use our package are presented here.
4. As an r‐package, *“TreeSimGM”* allows flexible and powerful stochastic phylogenetic tree simulations. Moreover, it facilitates the pipelining of outputs or inputs with other functions in r. *“TreeSimGM”* contributes to the tools available to the r community in the fields of ecology and evolution, is freely available under the GPL‐2 licence and can be downloaded at https://cran.r-project.org/web/packages/TreeSimGM.

## 1 INTRODUCTION

Macroevolutionary models generate phylogenetic trees resulting from speciation to extinction processes, starting from a single individual at the stem age of a clade or from two species at the crown age, that is the most recent common ancestor of the clade. The initial species evolve towards the present. Macroevolutionary models formalize and simplify real systems by generating phylogenetic trees reflecting the relationship between species and the overall number of species through time from assumed speciation and extinction processes. Each model has its particular assumptions and thus may give rise to different phylogenetic trees. Comparing these simulated trees to empirical trees stimulates new hypotheses regarding macroevolutionary processes (Alexander, Lambert, & Stadler, 2016; Bennett, Sutton, & Turvey, 2016; Gavryushkina et al., 2017; Hagen, Hartmann, Steel, & Stadler, 2015; Hey, 1992; Soul & Friedman, 2017) and allows deterministic forces to be identified (Harvey, May, & Nee, 1994; Pybus & Harvey, 2000; Stadler, 2013). We will now discuss the range of models under which we can simulate in “*TreeSimGM*.”

### 1.1 Yule model

One of the most popular phylogenetic models is the Yule model (Yule, 1924), where each lineage has the same constant speciation rate, which corresponds to an exponential waiting time between consecutive speciation events. Extinction does not occur.

### 1.2 Constant‐rate birth–death model (crBD)

The crBD model adds an explicit extinction process to the Yule model. This extinction process is analogous to the speciation process, with each lineage having the same constant extinction rate (Kendall, 1948; Mooers & Heard, 1997; Nee, May, & Harvey, 1994).

### 1.3 Bellman–Harris model (BH)

The BH model (Bellman & Harris, 1948) is an extension of the Yule model. The time to speciation for each lineage is modelled by the same arbitrary waiting time distribution rather than by an exponential distribution. As in the Yule model, extinction does not occur. Moreover, a BH model (under a symmetric speciation mode, see below), where species either speciate further directly after speciation or not at all, results in each tree on n extant tips being equally likely (Steel & McKenzie, 2001), also known as the proportional to distinguishable arrangements model (Aldous, 1996, 2001; Semple & Steel, 2003). A Weibull distribution of waiting time until speciation with the shape parameter tending to zero would lead to such a behaviour. Generality is an advantage of this BH model; however, this induces complexity and consequently poorly detailed analytical understanding (Hartmann, 2008).

### 1.4 Bellman–Harris with extinction model (BH‐ex)

The BH‐ex model is a BH model with extinction added. Thus, it generalizes the BH, crBD and Yule models. Each lineage has the same arbitrary waiting time distribution until extinction, independent from its speciation distribution. Such freedom when choosing distributions for the waiting times until speciation or extinction makes the BH‐ex a very flexible tool. For example, this model yields the Yule model when an exponential waiting time until speciation and no extinction are assumed, while the crBD model is obtained when an exponential waiting time until extinction is added. Age‐dependent models are BH‐ex models that use any species‐age‐dependent distribution for waiting times.

### 1.5 Models with lineage‐specific changes

Under the BH‐ex model, each new species is assigned waiting times to speciation/extinction from the same underlying probability distributions. Models with lineage‐specific rate changes relax the BH‐ex assumption that each individual event is described by the same given probability distribution. The lineage‐specific change models still assume one waiting time distribution for the time to speciation and one for the time to extinction. Descending new species can scale this distribution, though, with some factor *f*. In particular, each new species may change the waiting time to speciation or extinction with some probability *p*. If a change occurs for species i (with probability *p*), the waiting time is drawn from the speciation/extinction distribution and then scaled by some factor *f*_*i*_. This factor *f*_*i*_ is inherited by all descending species unless a further rate change occurs.

A shorter waiting time to speciation may occur through ecological traits, such as through the evolutionary acquisition of key characteristics by a certain lineage (also referred to as key innovations) (Hunter, 1998). Likewise, changes in the waiting time until extinction may be caused by changes in ecological traits. Additionally, such waiting time changes could be caused by the extinction of antagonists or by environmental changes (Simpson, 1953).

### 1.6 Symmetric and asymmetric speciation

**Figure 1:**
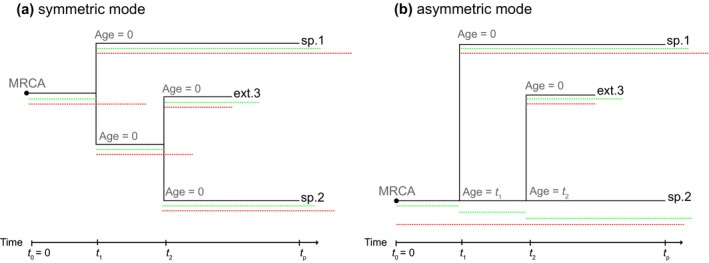
Model simulation for symmetric (a) and asymmetric (b) speciation modes. Speciation and extinction events are based on drawn waiting times from the distribution specified in the input. Drawn waiting times until speciation (green dashed lines) and extinction (red dashed lines) are plotted for each lineage, exemplifying the model mechanics. (a) Symmetric mode, where both descendants of a speciation event are new species with age 0. (b) Asymmetric mode, where one descendant of a speciation event is a new species and the second descendant is the mother species

Arbitrary waiting time distributions under BH‐ex in particular mean that the age of a species may influence the probability of speciation or extinction, depending on the chosen distribution. Furthermore, lineage‐specific changes occur only in new species, that is species of age 0. Thus, we need to specify the age of each species. We model two possibilities (see Figure 1 for a comparison): (1) symmetric speciation, where both species subtending a speciation event are considered new species with age 0, and thus, new waiting times to extinction are drawn while the mother species terminates (goes extinct); and (2) asymmetric speciation, where a speciation event results in one new species with age 0 and thus a new waiting time to extinction, while the second species inherits the age and the extinction time of its ancestor. Thus, under the asymmetric speciation mode, the “mother species” can undergo several speciation processes, as long as the drawn extinction time is not reached (Figure 1b), while a species terminates at a speciation event under the symmetric speciation mode.

**Figure 2:**
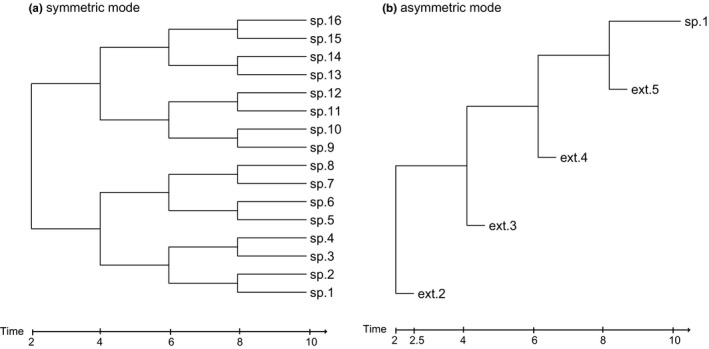
Distinct model behaviour under fixed waiting times of 2 Myr until speciation and 2.5 Myr until extinction for (a) symmetric and (b) asymmetric speciation modes

The relevance of the speciation mode is highly dependent on the model, that is the waiting time distributions and parameters. Under a Yule model or crBD, the two modes are equivalent, as each lineage at each point in time undergoes the same dynamics specified by the constant speciation and extinction rates. By contrast, Figure 2 shows the extremely different behaviours of the two speciation modes under a delta distributed waiting time to speciation and extinction, where speciation is set to always happen after 2 Myr and extinction is set to occur after 2.5 Myr.

### 1.7 Incomplete species sampling

Under any of these models, we allow for incomplete species sampling. We sample a number *N *×* frac* (*= n*) out of the final number of *N* extant species either uniformly at random or according to the pendant branch lengths; that is, longer branches are more likely to be sampled.

## 2 “*TREESIMGM”* PACKAGE

Although there are many speciation–extinction model simulation tools available (e.g. Herron, 2002; Höhna, May, & Moore, 2016; Maddison & Maddison, 2017; Rambaut, Grassly, Nee, & Harvey, 1996; Revell, 2012; Sukumaran & Holder, 2010; Webb, Ackerly, & Kembel, 2008), each tool is specific to a particular model class, or classes, and makes slightly different assumptions during simulations. Assumptions of when to stop a simulation, for example, vary. Common assumptions are as follows: (1) stopping once a specified stem (crown) age of a tree is reached, (2) stopping the first time a specified number of coexisting species has been reached or (3) conditioning the simulated tree on a specified number of coexisting species (Stadler, 2011). Under the last stopping assumption, the simulation is not stopped when a specified number of coexisting species is reached for the first time. Instead, a tree with many more coexisting species is simulated, and a random time is chosen when the specified number of coexisting species within the simulated tree is reached. Consequently, the resulting tree is the simulated tree stopped at this chosen time point. Different stopping assumptions complicate comparisons between models, thereby discouraging the user from exploring different models. In addition, these tools are implemented for different environments and platforms.

As far as we are aware, there is no implementation of the general BH‐ex model with lineage‐specific waiting time distributions available for simulating phylogenetic trees. Here, we present a flexible implementation of this model that allows simulations under a wide range of assumptions. The implementation for r is made available as an r‐package *“TreeSimGM,”* where GM stands for “general Bellman–Harris model”. With our implementation, it is possible to simulate phylogenetic trees based on a specified crown age (i.e. time since the first species arose in the clade) or the number of extant species. Moreover, this single implementation can simulate many different macroevolutionary models, given the nature of our lineage‐specific BH‐ex model with a symmetric or asymmetric speciation mode that takes any probability distribution for the waiting time until speciation and extinction.

Simulations start with one individual at the stem age of the clade. Speciation and extinction times are drawn from the user‐specified distributions. Whenever the extinction time is shorter than the speciation time, an extinction event happens. If the waiting time to speciation is shorter than that to extinction, a speciation event happens. Under asymmetric speciation, further waiting times to speciation for the mother species are drawn until the speciation time is greater than the extinction time. The ages of the new species are determined according to the speciation mode selected (for details see the “symmetric and asymmetric speciation” section). Simulations can also stop at a specified age (from a stem age until a specified time value) or number of extant species (for details see the “stopping assumptions” section).

The implementation presented in *“TreeSimGM”* allows to simulate general Bellman–Harris models with lineage‐specific shifts in speciation and extinction rate under incomplete sampling for the symmetric and asymmetric speciation modes using different stopping conditions, thereby facilitating pipelining and model behaviour testing, exploration and development (e.g. Hagen et al., 2015). Recently, an age‐dependent model with lineage‐specific shifts was suggested as a promising explanation for empirical tree imbalance (Holman, 2017). This study is an example of future research that can be conducted with the aid of *“TreeSimGM*.” Moreover, *“TreeSimGM”* enables the testing of speciation and extinction parameter inference methods (Alexander et al., 2016; Hagen, Andermann, Quental, Antonelli, & Silvestro, 2017; B.H. Warren, O. Hagen, F. Gerber, C. Thébaud, E. Paradis & E. Conti, unpubl.). Implementation input and output follows the r‐package *“TreeSim”* (Stadler, 2011), which allows simulation of rate shifts through time.

## 3 USING *“TREESIMGM”*

The number of simulated trees is defined by the parameter *numbsim,* and the simulation output is a list of phylogenetic trees in the *phylo* format (Paradis, Claude, & Strimmer, 2004). This format is the predominant format for phylogenies in r and is compatible with many other tree manipulation and statistics packages, avoiding thus the necessity of format conversion. All simulated trees are rooted and can be either ultrametric or non‐ultrametric, depending on whether the user desires to prune or not to prune the extinct lineages from the final tree. This pruning decision can be made by setting the parameter *complete* to *FALSE* (pruning) or *TRUE* (no pruning), where *TRUE* is the default.

### 3.1 Defining speciation and extinction distributions

The chosen distribution for the waiting time to speciation and extinction (parameters “waitsp” and “waitext,” respectively) is set just as one would invoke the random generator function in r, for example “*rweibull(0.5,1)*”, *“rgamma(1.5,1,2)*”, *“rexp(0.9)”*. The parameter of the chosen distribution depends on the order imposed by the random generator of the chosen distribution in r, skipping the first parameter that sets the number of observations. In cases of uncertainty, for example, regarding the Weibull distribution, invoking

*> ?rweibull*

returns, amongst other information:

*rweibull(n, shape, scale = 1)*

Therefore, we know that, for a Weibull distribution, setting the distribution parameter “waitsp” or “waitext” to “*‘rweibull(0.4)’*” tells “*TreeSimGM”* to use a shape of 0.4 and the default scale of 1. When *“‘rweibull(0.4,3)’”* is input, for example, shape is set to 0.4 and scale is set to 3. More generally, one can use any function returning a single waiting time per call.

### 3.2 Stopping assumptions

“*TreeSimGM*” has two main functions that simulate trees with a specified number of extant species (“*sim.taxa”* function) or a specified total age (*“sim.age”* function). With the function *sim.taxa*, the user can define the number of extant tips (*n*) in the simulated tree. To avoid always returning a tree in which *n* coexisting species are reached for the first time, potentially biasing simulations towards younger trees, the general sampling approach (Hartmann, Wong, & Stadler, 2010) is implemented. To use this approach, the parameter *gsa* needs to be *TRUE* and a number of coexisting species (*m*) with *n≪m* needs to be specified. A tree with *m* coexisting species is then simulated, and a random subtree with *n* coexisting species is chosen, producing random trees with *n* extant species (Hartmann et al., 2010). For simulations without the general sampling approach (*gsa=FALSE,* i.e. when the simulation is stopped, the first time that *n* coexisting species are reached), no value for *m* is needed.

The following code simulates 100 phylogenies with 20 extant species under a Yule model (i.e. exponential waiting times until speciation) with a speciation rate of 0.5:

*> yule <‐ sim.taxa(100, n=20, waitsp=“rexp(0.5)*”)

Note that when an extinction distribution is not specified, no extinction is assumed. Using the function *sim.age*, the user can define a final age for the simulated trees. The outputs of these functions will be either a phylogenetic tree with the desired age (from stem age until the present), 0 (zero) if the tree goes extinct before the defined age or 1 (one) if there is only one living species to be returned and *complete* is set to *FALSE*.

The following code simulates 100 phylogenies with an age of 4 Myr under a crBD (i.e. exponential waiting times until speciation and extinction) with a speciation rate of 0.9 and an extinction rate of 0.1:

*>crbd <‐ sim.age(100, age=4, “rexp(0.9)”, “rexp(0.1)”)*

Most of the examples below produce trees on n extant tips; however, the same logic is valid for simulations of trees with a fixed age, except regarding the specifics mentioned above.

### 3.3 The symmetric and asymmetric speciation modes

By default, simulations are done under a symmetric speciation mode. The following code is used to simulate trees with 15 extant species for a general model with an age‐dependent speciation process (Weibull waiting times with shape of 0.4 and scale of 3) and no extinction under an asymmetric mode:

*>gm_agesp_noext_asym <‐ sim.taxa(100, n = 15, waitsp = “rweibull(0.4,3)”, symmetric=FALSE)*

The following code is used to simulate a tree under the symmetric mode with the same speciation process, number of trees and number of extant species as in the previous example, and with and an exponentially distributed waiting time to extinction with parameter (i.e. rate 0.5) (one does not need to declare *symmetric* = *TRUE* because this is the default setting):

*> gm_agesp_costext_sym <‐ sim.taxa(100, n = 15, waitsp = “rweibull(0.4,3)”, waitext = “rexp(0.5)”)*

### 3.4 Lineage‐specific changes

**Figure 3:**
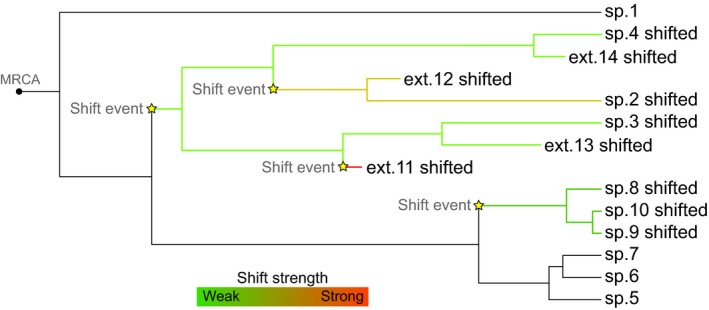
Simulated tree with changes in expected waiting times until extinction. Yellow stars mark where changes happened, and the strength of each simulated change is colour‐coded. Weak shifts scale the waiting time with a value close to one, and strong shifts scale the waiting time with a value much larger or smaller than one. The hereditary nature of the changes is evident on the scheme, where all descendant lineages, except those that underwent another change, inherit the previous scaling value

The probability of scaling the waiting time to speciation and extinction (shown as stars in Figure 3) is defined by the parameters *“shiftsp*$*prob” and “shiftext*$prob” for speciation and extinction changes, respectively. When a new species is formed (one new species subtends an asymmetric speciation event, two new species subtend a symmetric speciation event), the new species obtains a scaled waiting time to speciation with probability “*shiftsp$prob”* and a scaled waiting time to extinction with probability *“shiftext$prob*.” By default, these probabilities are zero, meaning that no change will occur. If the probability for a change is not zero, the waiting time is determined by picking a waiting time from the distribution for speciation/extinction and scaling this waiting time with factor *f* (see Figure 3). The user must provide the distribution or function from which the scaling factor *f* is defined, in the same way speciation and extinction are set.

The following code is used to simulate Yule trees (speciation rate of 0.8) with shifts happening with a probability of 9% and a scaling factor *f* chosen uniformly from within [0.5, 1.5]:

*>yule_shiftsp <‐ sim.taxa(100, n=15, waitsp=“rexp(0.8)”, shiftsp=list(prob=0.09, strength=“runif(0.5,1.5)”))*

For every node, speciation and extinction scaling factors are stored at “shiftsp” and “shiftext,” respectively. Additionally, extant and extinct tips with shifts upon speciation are marked with “1” (shift) or “0” (no‐shift) under “shifted.sp.living” and “shifted.sp.extinct,” respectively. The “shifted.ext.living” and “shifted.ext.extinct” follow the same system but store scaling factors in the extinction process. The nodes and the strengths of the scaling factors of the sixth simulated tree from the example above are retrieved with the following code:

*> yule_shiftsp[[6]]$shiftsp*

### 3.5 Cross‐validation with other existing models

**Figure 4:**
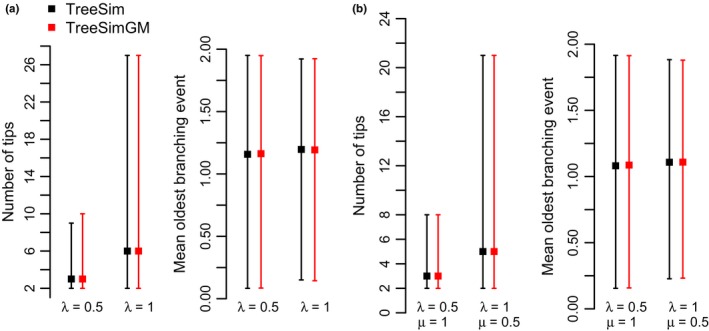
Comparison of *“TreeSim”* and *“TreeSimGM”* (a) Yule and (b) Constant‐rate birth–death (crBD) models with two distinct parameters. Comparisons are based on 100,000 simulated trees, where extinct trees were excluded from the analysis. Median and 95% confidence intervals of the number of extant tips and the mean oldest branching events are plotted in black for *“TreeSim”* and in red for *“TreeSimGM”*

To validate our implementation regarding the Yule and crBD models, we compared the number of extant tips and the mean oldest branching event from 1,000 trees simulated with “*TreeSim*” and “*TreeSimGM*” under the same conditions (Figure 4).

We provide further validation examples inside the “*TreeSimGM*” documentation (help and vignette), in particular we provide a validation of a model with constant speciation and age‐dependent extinction specified by a gamma‐distributed waiting time, where we simulate trees and estimate the true parameters from an independently implemented tool (Alexander et al., 2016).

## 4 CONCLUSION

We present here a flexible simulation tool implemented in r that unifies some existing simulation methods into one framework, that is a general Bellman–Harris model with or without lineage‐specific changes under symmetric or asymmetric mode that can be simulated for a certain age or number of extant tips in a tree.

*“TreeSimGM”* facilitates pipelining of outputs and/or inputs, model behaviour testing, exploration and development, and analysis of the performance of existing and future inference methods.

## CONFLICT OF INTEREST

The authors gave final approval for publication and have no conflict of interest to declare.

## AUTHOR CONTRIBUTIONS

O.H. and T.S. contributed equally and critically to the r‐package and to the drafts of this article.

## DATA ACCESSIBILITY

All data used in this article were simulated with *“TreeSimGM*” and r‐package freely available under the GPL‐2 licence at CRAN. The r‐package, a reference manual and a vignette are available at https://cran.r-project.org/web/packages/TreeSimGM.
